# Use it or lose it? Identifying reasons for the low use of psychosocial support by hospital staff

**DOI:** 10.1186/s12960-023-00830-8

**Published:** 2023-06-09

**Authors:** Frank van de Baan, Lieze Poesen, Daan Westra, Bram Fleuren, Dirk Ruwaard, Fred Zijlstra, Rachel Gifford

**Affiliations:** 1 Department of Health Services Research, Care and Public Health Research Institute (CAPHRI), Faculty of Health, Medicine and Life Sciences, Maastricht University, 6229 GT Maastricht, The Netherlands; 2 Department of Work and Social Psychology, Faculty of Psychology and Neuroscience, Maastricht University, 6229 ER Maastricht, The Netherlands

**Keywords:** Well-being, Mental health, Healthcare, Healthcare workers, Organizational support, Mixed-methods, Crisis

## Abstract

**Background:**

Psychosocial support programs are a way for hospitals to support the mental health of their staff. However, while support is needed, utilization of support by hospital staff remains low. This study aims to identify reasons for non-use and elements that are important to consider when offering psychosocial support.

**Methods:**

This mixed-method, multiple case study used survey data and in-depth interviews to assess the extent of psychosocial support use, reasons for non-use and perceived important elements regarding the offering of psychosocial support among Dutch hospital staff. The study focused on a time of especially high need, namely the COVID-19 pandemic. Descriptive statistics were used to assess frequency of use among 1514 staff. The constant comparative method was used to analyze answers provided to two open-ended survey questions (*n* = 274 respondents) and in-depth interviews (*n* = 37 interviewees).

**Results:**

The use of psychosocial support decreased from 8.4% in December 2020 to 3.6% by September 2021. We identified four main reasons for non-use of support: deeming support unnecessary, deeming support unsuitable, being unaware of the availability, or feeling undeserving of support. Furthermore, we uncovered four important elements: offer support structurally after the crisis, adjust support to diverse needs, ensure accessibility and awareness, and an active role for supervisors.

**Conclusions:**

Our results show that the low use of psychosocial support by hospital staff is shaped by individual, organizational, and support-specific factors. These factors can be targeted to increase use of psychosocial support, whereby it is important to also focus on the wider hospital workforce in addition to frontline staff.

## Background

A healthy hospital workforce is essential for the functioning of hospitals and the wider health system [1–4]. However, around the world a large proportion of hospital staff face mental health burden [5–11]. In part this stems from psychological suffering at work, such as having to deal with high levels of uncertainty, exhaustion, a perceived loss of control, or traumatic events [6–8, 12, 13]. Psychosocial support is recommended as an important intervention to help staff deal with such suffering [13–16], and is typically defined as a range of services offered by mental health professionals within the organization to staff with pressing need [17]. Most hospitals typically have some form of psychosocial support in place for this aim [18–21, 23, 24]. Yet, studies continue to show that while there is a need [22, 23, 25, 26], the actual use of these services by staff is remarkably low [18–21, 23]. Therefore, it is important to study the factors that determine non-use of psychosocial support, especially during times of high need.

Previous studies found several individual level factors that keep hospital staff from using psychosocial support within the hospital. Such factors include a personal tendency to solve adverse mental health outcomes individually [19, 27], or an overall reluctance to seek professional help [19, 20, 28]. However, recent systematic literature reviews highlight the need for empirical research, stating that most studies were of poor quality [19, 29, 30]. In addition, the majority of factors were identified through quantitative studies, which do not lend themselves for a comprehensive exploration of experiences [31, 32]. Moreover, qualitative studies focused only on part of the hospital workforce, namely frontline staff (e.g., staff providing care to COVID-19 patients) [28]. This presents an additional gap given that other hospital workers, such as support staff and management, can experience mental health issues as well [18, 33–35]. Studies have shown low utilization of psychosocial support among non-frontline staff as well [21], although the reasons for non-use remain unknown.

To further explore this issue, this paper presents a mixed-method multiple case study to examine hospital staff’s reasons for non-use of psychosocial support during a time of arguably high need, namely the COVID-19 pandemic. The present study draws upon survey data and in-depth interviews with hospital staff to identify: (1) reasons for non-use of psychosocial support offered by the hospital during the COVID-19 pandemic, and (2) elements deemed important by hospital staff regarding the offering of psychosocial support within the hospital. As a result, this study provides insights into the diverse support needs of staff across different functions, and potential ways to increase support effectiveness. Moreover, our findings go beyond the analysis of individual factors, including broader factors that can play a role, such as organizational aspects and features of the support itself. Thereby, this study facilitates hospitals in mitigating mental health issues of staff.

## Methods

This study is part of a larger 2-year research project investigating hospitals’ responses to the COVID-19 crisis and their effects on employees’ sustainable employability. In line with the American Psychological Association, we define ‘psychosocial support’ as services that address mental health issues (i.e., psychological and emotional issues that affect mental and social well-being), for example, through mental health counselling, psychoeducation, peer support, or spiritual support [17]. It is offered by mental health professionals such as psychologists, social workers, and pastoral counsellors [17]. This project was granted ethical approval (FHML-REC/2020/110) and the study follows the consolidated criteria for reporting qualitative research (COREQ) [36].

### Data collection, materials, and participants

For this paper, we collected survey data and in-depth interview data from hospital staff. The surveys collected both qualitative data via open questions and quantitative data. Staff from four different hospitals located in one province of the Netherlands participated in the study.

### Surveys

For survey distribution, the hospitals provided us with the e-mail addresses of all their hospital employees (*n* = 18,853). Via direct e-mails and messages on the internal websites of the hospitals, the 18,853 hospital staff members were invited to participate in the survey. The survey was sent out on four measurement occasions in December 2020, March, June, and September 2021. Participation was voluntary and informed consent was obtained from all participants. Respondents were not obliged to fill out all questions and could stop with the survey at any given time without providing a reason. A more detailed description about the entire survey study is outlined elsewhere [37]. Ultimately, 1514 hospital staff members participated in the survey. The demographic and occupational characteristics of the 1514 survey participants can be found in Appendix 1.

We first asked survey participants whether they had received support from the psychosocial team within their hospital, to which participants could answer ‘yes’ or ‘no’. We used this first question to quantitatively assess whether the degree of use of psychosocial support among the staff of participating hospitals was comparable to that of other studies. More specific to the two main research questions, surveys contained two open-ended questions regarding psychosocial support utilization that were analyzed qualitatively. The first open-ended question was given to all survey participants, and asked “Do you have any suggestions or advice for the psychosocial support team within your hospital?”. In addition, a second question was presented to participants who indicated that they did not make use of psychosocial support (i.e., answering ‘no’ to the first psychosocial support question), “Have you considered making use of support from the psychosocial team within your hospital?”. If participants indicated ‘yes’ to this question, the respondents were asked to write their reason(s) for non-use in an open field. In total, 274 of the 1514 survey respondents provided 418 unique answers to the two open-ended questions regarding psychosocial support utilization. The occupational characteristics of these 274 survey respondents can be found in Table 1.

**Table 1:** Occupational characteristics of the qualitative sample (274 survey respondents and 37 interviewees): amount of staff per function and case (hospital)

Staff function	Case 1		Case 2		Case 3		Case 4		Total
	Survey*	Interview	Survey	Interview	Survey	Interview	Survey	Interview	
Manager	9	1	2	0	3	4	1	0	20
Medical specialist	6	2	4	1	6	2	0	0	21
Physician resident	2	3	1	2	3	0	0	0	11
Nurse	24	3	22	0	39	9	21	3	121
Support staff	42	0	26	0	43	7	19	0	137
Total	84	9	55	3	94	22	41	3	311*

*For one respondent the function type was missing

### In-depth interviews

For the in-depth interviews, we used purposive sampling. We requested a contact list from the hospitals that included representative individuals across all levels of the organization (i.e., managers, medical specialists, residents, nurses, and support staff). Recruitment started in March 2021. To recruit extra interviewees, at the end of the third survey (i.e., June 2021) participants were informed about the possibility to participate in an interview and were given contact details of the researchers. In addition, this information was posted on the internal staff website of one of the participating hospitals. Recruitment of new participants stopped when data saturation was reached. Interviews were conducted in English or Dutch by a native English (RG) and Dutch (FB) speaker, and lasted on average 60 min. Interviews were recorded and transcribed verbatim. Interviews took place in-person or digitally, in light of then applicable COVID-19 regulations. For each interview, the lead interviewer created a written summary of the main findings immediately after the interview and interviewers took extensive notes during the interview. All interviewees were given the written summary to check whether it resonated with their interview experience, after which two interviewees requested minor changed to be made. All interviewees were also offered the opportunity to member check their transcript. No interviewee requested changes to be made to the transcript, such as the deletion of certain sections. Informed consent was obtained from all interviewees. Translations were performed by FB where necessary. As part of a larger project, interviews covered a variety of topics such as experiences of working during the crisis, group dynamics, and workload. The interviews included a subset of questions regarding the psychological and emotional effects of working during the crisis (e.g., ‘How has your health and well-being at work been affected by COVID-19 and the adaptations in your hospital?’) and their thoughts on, and use of, psychosocial support (e.g., ‘What did you think of the psychosocial support that was being offered in your hospital?’). In total, 36 in-depth interviews (one of which included a duo-interview with two nurses simultaneously) were conducted between April and August 2021. The occupational characteristics of the 37 interviewees can be found in Table 1.

### Data analysis

For the quantitative analysis, descriptive statistics were used for the first survey question to identify the frequency of psychosocial support use across the four measurement occasions of the survey.

For the qualitative analysis, we used the qualitative data collected through the 418 answers on the two open-ended questions in the surveys and the 36 in-depth interviews. We used the constant comparative method to analyze the answers of both sets of data (i.e., the open-ended questions of the surveys and the interview data) [38, 39]. This qualitative analysis consisted of three phases. First, both sets of data were analyzed separately with FB inductively coding the in-depth interviews and LP inductively coding the open-ended question responses of the surveys. During this process, code lists and emerging themes were shared in group discussion between FB, LP, and RG. In the second phase, when initial coding was completed, FB and LP went back to their datasets to group codes into main themes, constantly going back and forth through data, seeking similarities and differences among the many codes and critically assessing whether codes could be grouped into common themes. During this process, emerging insights were shared and discussed with the total research group (seven researchers in total)—who could provide a more critical, outsider perspective—until analysis was completed [40]. Third, upon completion of the process, the derived themes were used to create a codebook (see Appendix 2). When finalized, the codebook was used to assess intercoder reliability, whereby FB and LP independently coded 78 answers to the open-ended questions (18.6% of total open-ended questions), and four in-depth interviews (11.1% of the total amount of interviews). Krippendorff’s Alpha was calculated using Atlas.ti version 9.0 with the score being 0.91, indicating high agreement [41].

## Results

We first quantitatively calculated the use of psychosocial support within the hospital across the four measurement occasions of the surveys. We identified the usage to be the highest during the first measurement occasion in December 2020, at 8.4%. Usage declined over time, with 3.6% of the respondents using psychosocial support in September 2021. The results of psychosocial support usage across the four surveys are presented in Table 2.

**Table 2:** Frequency of psychosocial support use per measurement occasion of total survey participants

Survey	*n* (%)		
	Yes	No	Total
One (December, 2020)	77 (8.4%)	838 (91.6%)	915 (100%)
Two (March, 2021)	49 (5.1%)	903 (94.9%)	952 (100%)
Three (June, 2021)	27 (4.4%)	585 (95.6%)	612 (100%)
Four (September, 2021)	18 (3.6%)	489 (96.4%)	507 (100%)

### Reasons for non-use psychosocial support

Next, in light of our first study aim we analyzed the interview data and answers to the open-ended survey question ‘’what are your reason(s) for non-use of the psychosocial support offered by the hospital?’’. Four main themes emerged from the qualitative data: (i) Unnecessary, (ii) Unsuitable, (iii) Unaware, and (iv) Undeserving. Within these themes, we found further subthemes that we detail below. Additional quotes for each theme and their subthemes can be found in Table 3.

**Table 3:** Quotes on reasons for non-use of psychosocial support offered by the hospital

Theme	Subtheme	Quote (Q = quote number, staff function, C = case number, data source)
Unnecessary	Not needed	“No, I didn't use that supported at all. I, I, I know what it was there, but I just didn't need them. For me fortunately, I can leave my work at work.”—Q1, Medical specialist, C3, interview
		“I deemed my complaints to be normal considering the hectic at work in combinations with the situation at home.”—Q2, Support staff, C2, survey
	Other support	“At some point it was often pointed out like: “yeah the psychosocial support team is here, the palliative team is here, if you ever want to talk to someone, or social work.” But every time we had those talks we thought: actually it’s much better if we just uhm, evaluate in our own team and discuss whether there are certain things people want to talk about.”—Q3, Nurse, C3, interview
		“I thought this was something I had to learn to deal with myself.” – Q4, Nurse, C1, survey
		“There were other institutions that I could approach for my care needs.”—Q5, Management, C2, survey
Unsuitable	Not helpful	“I believe it won’t have much effect.”—Q6, Nurse, C4, survey
		“At the time I didn’t see the added value in it and with some members of the BOT team I don’t have a click.”—Q7, Support staff, C1, survey
	Type of support	“When it was needed, it was only possible to do it online instead of face-to-face.”—Q8, Support staff, C3, survey
		“[I] work a lot during the night and weekends, then there is no support available.”—Q9, Nurse, C2, survey
	Lack of time	“I didn’t make time for it.”—Q10, Nurse, C2, survey
		“I wasn’t being given the time or space to think about making an appointment or giving a call.”—Q11, Nurse, C2, survey
Unaware	Not sufficiently actively promoted	“I wasn’t aware of the existence of this team. For me personally I found out to late!!”—Q12, Manager, C1, survey
		“During the first wave they sometimes visited and asked how you were doing. In the second wave, when I had corona myself, I actually didn’t really know where I could go to and what I then could say or ask.”—Q13, Physician resident, C1, survey
		“I need this [psychosocial support]. Last week I went looking for the phone number, because during the beginning of the corona period it was communicated that everyone could call. The only thing I was now able to find was the working conditions service, and then the threshold is too high. So now I need help but cannot get it.”—Q14, Support staff, C1, survey
Unaware	More than COVID-19	“It would be nice to have such support also for non-COVID related stuff… Clearing your mind without having to go through an entire trajectory.”—Q15, Support staff, C2, survey
		“Yes I came across it, that there was a living room where you could go to. Well, I really interpreted that as that being there for the people in the frontline. And I was not onsite so I, I never felt like that was addressed to me. And yes looking back I may have had the need to have a little extra talk or to be taken care of.”—Q15, Support staff, C3, interview
		“Of course, people working from home also had quite some fears about COVID and stuff…. some really, really struggled just having to be home all the time and not talking to people, not seeing people, having difficulties concentrating at home. […] And from accounts I heard, they didn't feel supported.”—Q16, Support staff, C3, interview
Undeserving	Others need it more	“There were- there were phone numbers and on internet. You know, a lot of explanations going on… The blogs and- and that you could call if- if you wanted to talk. But it was, I think, not for me but for the nurses because they were- they were at the bedside where patients died. So, I guess, although I felt depressed the first few weeks, I- I didn't want to call that number because I felt it was nothing compared to what the nurses have to go through.”—Q21, Support staff, C3, interview
		“For me there is also a barrier because I think colleagues on the specific corona department should get priority.”—Q18, Support staff, C3, survey
		“There is no one in the team of which I think: that person won’t take it anymore. […] But I think that that is for example different for the IC. […] and I think for the wards sometimes as well. […] During the first wave you had of course a lot [of patients] at the ward who were really sick but didn’t go to the IC. If you then have shift and four people die, yes that is of course not, not a nice shift.”—Q19, Physician resident, C2, interview
		“Yes actually, yes it was.. I wouldn’t be right if I said it was a piece of cake. […] [But] we’re a surgical ward, we are used to hectic situations and fluctuations in busyness. It’s either very busy or very quiet,, we’re used to that.” [But] the new colleagues from the paediatrics ward [who helped out on the COVID ward], for them I think it is really challenging.—Q20, Nurse, C1, interview

All quotes are from different participants

*(i) Unnecessary*. One of the key reasons that emerged from the data regarding non-use, was that psychosocial support offered by the hospital was deemed as unnecessary by the respondents. In this theme, we identified two subthemes: ‘not needed’ and ‘other support’. Individuals indicated that for themselves, support was not (yet) needed or not needed anymore (‘not needed’) [quotation 1 and 2 (Q1; Q2) in Table 3]. It is noteworthy to point out that some individuals shared that although at first they did not deem support necessary, looking back they realized that receiving psychosocial support would have been beneficial.

“During the moment itself it doesn’t seem to be necessary. But looking back, that’s a different story.”—Medical specialist, Case 2, Survey.

Other staff deemed support from others, such as friends or family, or self-support as sufficient or better suited for coping with the crisis (‘other support’) (Q3; Q4; Q5).

*(ii) Unsuitable*. This second theme included staff that felt the need for psychosocial support offered by the hospital, but ultimately did not use it, because they deemed it unsuitable. Staff mentioned they felt the support that was offered would not be effective (Q6; Q7), or was perceived to have negative connotations, subcategorized as ‘not helpful’. Among the negative connotations mentioned were a perceived lack of confidentiality, or fears of stigma. As one survey respondent indicated:

“I don’t want to appear weak.”—Support staff, Case 2, survey

Another subtheme, ‘type of support’, included staff not using psychosocial support because of how the support was being offered (e.g., preferring face-to-face instead of online) (Q8), or the moments at which it was offered (e.g., not offered during the weekend) (Q9). As a third subtheme, ‘lack of time’ was described by staff as a further aspect for deeming psychosocial support unsuitable. Staff were either not being given time during working hours to make use of psychosocial support, or they did not take the time themselves (e.g., due to being too busy) (Q10; Q11).

*(iii) Unaware*. The next main theme we identified in the data was ‘unaware’. This can be seen as the lack of awareness of the availability of psychosocial support among staff. In the subtheme ‘not sufficiently actively promoted’, staff reported that they did not know psychosocial support was available for them to use (Q12). Moreover, some staff reported that, while they were aware of there being some sort of psychosocial support available, they were unaware on how or when the psychosocial support team could be reached (Q13; Q14). We identified ‘more than COVID-19’ as another subtheme. Some staff were under the impression that psychosocial support was only related to COVID-19 issues, while they were in need for support for other issues (Q15). Besides that, many of the respondents not (directly) involved in COVID-19 care perceived the support to not be intended for them. Staff commented on the fact that even though they also experienced difficulties due to the pandemic, they felt that the hospitals’ attention towards them was insufficient (Q16; Q17). As one participant stated:

“Have attention for the deaths on non-COVID departments. More people are dying here now as well compared to the normal situation, I notice that especially nurses are struggling with this.”—Physician resident, Case 2, survey

*(iv) Undeserving*. Despite acknowledging the value of psychosocial support, some staff did not use the support, because they assumed others to be in a worse position, and thus in greater need or more deserving of support than them (‘others need it more’) (Q18). In other words, they felt ‘undeserving’. In interviews, some staff compared their own experiences and mental status to that of others, whereby they often identified a different department or function type that, in their opinion, would be worse off:

“There were phone numbers and on [the] internet, you know, a lot of explanations going on. [Stating] that you could call if you wanted to talk. But it was, I think, not for me but for the nurses because they were at the bedside where patients died. So, I guess, although I felt depressed the first few weeks, I didn't want to call that number because I felt [that] it was nothing compared to what the nurses have to go through.”—Support staff, Case 3, interview.

This was not only the case for support staff or management, but also those working in the frontlines. For example, during interviews staff from the emergency care department deemed staff from the nursing wards to be more in need of support, because they faced more patient deaths (Q19). Similarly, staff from the nursing wards deemed staff from other specialties who helped temporarily to be more in need of support, because they were less used to the hectic situation (Q20).

### Important elements regarding the offering of psychosocial support

For the second study aim, we analyzed the interview data and the answers to the survey open-question “Do you have any suggestions or advice for the psychosocial support team within your hospital?”. Overall, we identified four themes: (i) after the crisis, (ii) accounting for diverse needs, (iii) accessibility and awareness, and (iv) active supervision. Additional quotes for each theme can be found in Table 4.

**Table 4:** Quotes on important elements regarding the offering of psychosocial support by hospitals

Theme	Quote (Q = quote number, staff function, C = case number, data source)
After the crisis	“There should be structural attention for this!”—Q21, Medical specialist, C2, survey
	“What they did well already during the first peak, is the taking care of professionals. Then they really created teams for psychosocial support […] And then afterwards we also had a digital meeting to see how we could keep doing that structurally. But I didn’t hear anything from that ever since.”—Q22, Nurse, C3, interview
	“I think this team has since been disbanded, so in that case it would be nice if there was an alternative place where you can go for psychosocial care. If there is, there is too little known about it.”—Q23, Nurse, C3, survey
Accounting for diverse needs	“On the one hand lower the threshold more than currently is happening and on the other hand make it more anonymous. Support together with peers is nice, but on the other hand it is sometimes nice to be able to talk with a stranger.”—Q24, Support staff, C3, survey
	“I miss an independent person who you can go to.”—Q25, Nurse, C4, survey
	“Especially ask the employee what he/she thinks he/she needs, really listen to that and where possible also actually accommodate that.”—Q26, Support staff, C1, survey
Accessibility and awareness	“Create more awareness as to where people can go to and make this especially easily accessible. If I don't feel well and run into problems, scouring the intranet for the appropriate contact details is not what I will do. However, if I can walk-in somewhere for a first talk, I will probably schedule an appointment.”—Q27, Support staff, C3, survey
	“Make it more easily findable on Intranet.”—Q28, Medical specialist, C3, survey
	“Psychosocial support is at this moment voluntarily, while I think it would be could to have a more ‘mandatory’ reflection on difficult cases or situation and what it did to you more often.”—Q29, Management, C2, survey
	“Nurses think they can solve everything for themselves and often go beyond their limits. [They] feel the work pressure at the department and the requests regarding scheduling work, want to go home after a long shift which makes that they ask for support too late. Provide unsolicited psychosocial support and approach employees personally with regard to feeling safe.”—Q30 Nurse, C2, survey
Active supervision	“Perhaps do it via supervisors so that there is time during work to pay attention to this. Now there often is ‘no time’ or colleagues or the supervisors see it as weird/abnormal if you go there.”—Q31, Physician resident, C1, survey
	“It is well organized, but the one who needs support normally doesn’t search and asks for it themselves. Supervisors have an important role, they hear [and] feel what and whether something is going on with an employee.”—Q32, Support staff, C3, survey
	“Provide support to supervisors so that they can better help their employees. They are often the first point of contact in case of problems.”—Q33, Support staff, C3, survey

All quotes are from different participants

*(i) After the crisis.* All individuals, despite their use or non-use, recognized the importance of support being offered. Staff described how the need only arose after the height of the crisis:

“In my experience, the consequences of the COVID pandemic are only now really starting to become clear. […] Exhaustion, [feeling] emotionally overloaded, [these] are things that I recognize in myself and in colleagues, even though everyone has had a holiday. [Psychosocial support teams should] now go to departments to be able to do something for the employees here.”—Support staff, Case 2, survey.

In addition, staff highlighted the utility of psychosocial support beyond crisis times (e.g., after an emotionally demanding day at work) (Q21). However, offering psychosocial support more structurally seems to not have been properly addressed by the participating hospitals, with staff stating that psychosocial support team were disbanded (Q22; Q23). Moreover, some staff were unable to reach out to the psychosocial support team after the second COVID-19 wave (i.e., December 2020) (Q14).

*(ii) Accounting for diverse needs*. The hospital staff provided numerous suggestions regarding offering different types of psychosocial support, overall signaling a need to diversify. Staff provided suggestions regarding the composition of the psychosocial support team (e.g., including social workers, spiritual counsellors, peers, or only psychiatrists/psychologists) and regarding how the support should be offered (e.g., over the phone, face-to-face, or online) (Q24; Q25). Moreover, staff proposed various ways of promotion (Q26). One manager, for example, suggested creating open hours for employees to drop in:

“Create an open consultation hour where employees can go for a quick question regarding coaching/counselling in these times.”—Management, Case 3, survey.

*(iii) Accessibility and awareness*. Staff highlighted the importance of ensuring that psychosocial support is easily accessible and that hospital-wide awareness is created. They often mentioned that they perceived barriers to accessing the psychosocial support team which, coupled with the general tendency to not proactively seek help, prevented them from getting support (Q27; Q28; Q29; Q30). One nurse, for example, stated:

“The threshold for contacting the [psychosocial support] team is way too high. It has already been stated several times to the team that they must approach all employees personally. Nurses often have the tendency to put themselves in the background and will therefore experience a barrier to seek help. If you contact people personally this can lower the threshold.”—Nurse, Case 4, survey.

*(iv) Active supervision*. As a final important element, staff highlighted the key role of supervisors. Since supervisors or team leaders have close ties to their staff, staff commented on the active role team leaders (can) play in monitoring mental well-being of their team. Various suggestions were provided on how supervisors can implement this, for example, by regularly checking up on their personnel, making them aware of the possibility for psychosocial support, and identifying other needs of their staff (Q31; Q32).

“[A good team leader is] always there for us. And always asking us: how are we doing and what are we struggling with? What can be changed? What can we do differently? Um yeah that, that’s really, really so important to… To survive.”—Nurse, Case 1, interview.

In order to be able to play an active role, staff suggested that the hospital should support the team leaders in obtaining the necessary skills to detect the need for psychosocial support (Q33).

## Discussion

This study aimed to examine the reasons for non-use of psychosocial support by hospital staff, and to identify the elements that they deem important regarding the provision of such support within the hospital. In line with previous studies, we found support use to be low [18, 19, 21], whereby we found that staff deeming support unnecessary, unsuitable, being unaware of the availability of support, or feeling undeserving of psychosocial support contributed to low use. Overall, this is problematic considering that neglecting the well-being of staff can exacerbate mental health issues and threaten overall hospital functioning [1–3]. We, furthermore, found that long-term support, accounting for diverse needs, increasing accessibility and awareness, and active supervision were deemed important elements for offering adequate psychosocial support. Extending previous work that mainly looked into individual level factors [19, 20, 27], our study shows that low use can more profoundly be explained through the combination of individual, organizational, and support-specific factors. Our results further show that these themes are not unique to frontline workers, but are also present among the wider hospital workforce.

Regarding individual factors, low use seems to stem from a lack of recognition of symptoms of adverse mental health outcomes, of the benefits of psychosocial support, and of the individual need for this type of support. Indeed, studies conducted among frontline staff early in the pandemic found that they have a tendency to view their problems as not severe enough [18, 19]. Next to that, focusing on self-reliance or other ways to deal with adverse mental health outcomes, and the idea that others are in greater need of support, can play a role. Individual factors may likely stem from the fact that workplace mental health is subject to normative pressures (i.e., expectations and norms that shape individual behavior), including the views that mental health issues are abnormal, should be dealt with individually, and that those with mental health issues should not work [42, 43]. This can make discussing mental health issues difficult [42, 43]. When dealing with mental health issues, hospital staff may worry that coming forward about mental issues could have negative consequences including social exclusion or being seen as weak or less competent [43, 44]. Such effects have been evidenced, both within and outside of healthcare settings [42]. For those in leadership positions, normative pressures may play an even bigger role given their position, whereby they are expected to show strength and resilience [45].

Concerning organizational factors, a lack of active promotion of psychosocial support can play a role. The fact that hospital staff indicated they were in need for psychosocial support but ultimately did not make use of it because they were unable to find the support is striking. We know from literature on wellness interventions, such as exercise or resilience programs, that employees tend to lack awareness of organizational programs and interventions in place due to a lack of communication by the organization and overlooking it due to day-to-day tasks [46]. Our findings support this notion and, furthermore, show that even when communicated, a lack of clarity as to whom support is for can hamper the use. Employees perceived the support offered by their organizations to be directed primarily towards staff they deemed to be suffering most (i.e., those providing COVID-19 care). However, mental burden is present among all hospital staff members and not limited to crisis situations [23, 33, 34]. Researchers have called for greater consideration of non-clinical staff within hospitals given their importance for hospital functioning, and our findings support this notion [35].

Features of the psychosocial support program itself can also be a hampering factor for use of the support. Among others, whether the support is confidential or not, led by peers or professionals, or offered at specific times or via appointment can affect usage. Given the aforementioned sensitivity of the subject, whether the support is offered in a confidential one-on-one setting can increase the use of psychosocial support by some staff [47]. Conversely, psychosocial support within a group or team context could increase the use of psychosocial support by other staff. Research has shown that, if offered in a safe environment, group interventions were seen as valuable by staff [48, 49]. This regards, for example, psychosocial support offered in a setting, where hierarchical power structures are loosened through sharing challenges without a focus on problem solving but instead a focus on open dialogue and sharing experiences [48, 49]. Overall, different types of support seem to be accompanied by varying barriers for certain staff, affecting the usage of the support. Given time and financial constraints, it could be that hospitals do not deem offering a broad array of support a priority. Yet, interventions such as peer support, mindfulness training and stress management have all been shown to improve mental well-being and reduce long term absenteeism within hospitals while being of little expense to the organization [50]. Considering the significant impact of mental health issues on budgets alone, with absenteeism and attrition within health care costing the US 4.6 billion dollars per year [6], dedicating time and resources to offering long term psychosocial support in various forms can thus outweigh the costs.

### Implications

Our findings offer valuable insights for organizations regarding enhancing the use of psychosocial support. In the short term, hospitals should establish and sustain diverse psychosocial support interventions, along with regular communication on their availability and the significance of prioritizing mental health. These efforts should be targeted towards all employees. In addition, utility of support could be enhanced through two more long-term strategies.

Team leaders can play an important role given their intermediary position in the organization. Being in close contact with their staff, they are in the position to closely monitor staff well-being, create awareness, and refer those who potentially need support to the right channels. Research has, however, shown that team leaders can lack the necessary skills to do so, especially considering the sensitivity of the subject [52]. It can be a worthwhile strategy to train team leaders to acquire the necessary skills, including how to promote self-caring strategies, and how early signs of distress can be recognized [53].

Ultimately, hospitals and their staff would benefit from a culture where discussing mental health issues is normalized. This might be complex, as it requires stepping away from professional and social norms currently present in hospitals, such as a focus on high performance, self-reliance, and not burdening others [54]. In addition to the aforementioned normative pressures surrounding workplace mental health, this culture makes it particularly challenging for staff working in health care to disclose mental health issues, which in turn can exacerbate the issue [54]. However, fostering a caring work environment could serve as a possible solution [55]. This type of environment is characterized by a culture where, instead of avoiding difficult interpersonal interactions between colleagues, staff feel empathy for one another and take action when a colleague is in need for care [55]. This caring culture can be achieved through a process of infusion, sustainment, and replenishment [56]. Infusion includes screening for and hiring of candidates that show compassion. Sustainment entails the use of rewards and recognition for those who show compassion. Replenishment consists of the use of financial resources as means to assist staff with dealing with hardship, for example, to allow for paid time off. Overall, this three step process can help in legitimizing and encouraging the formation of a caring work environment [55, 56].

### Limitations

This study is subject to several potential limitations. First, the study was conducted in the Netherlands during a crisis period, which could affect generalizability of findings to other settings and contexts. However, given that the prevalence of use of psychosocial support in our sample is similar to that of studies conducted in other countries [15–20], and considering that research has shown that hospital work during regular circumstances can result in the need for psychosocial support as well [23], a certain degree of generalizability seems to be present. Second, our sample might be biased through a ‘healthy worker effect’ [57], whereby those with more severe mental problems might not be included in the sample due to absence at work or unwillingness to participate. This may mean that additional themes specific to this group were missed. Nonetheless, considering that participants who were included did state that they were in need of support, this bias may be limited. Third, while the study shows that hospital staff deem an active role for team leaders important, our data did not allow us to assess how supervisors themselves feel about taking on this role. Future studies are needed to tease out team leaders’ perspectives.

## Conclusion

Our results show that the low use of psychosocial support by hospital staff is shaped by individual, organizational, and support-specific factors. These factors can be targeted to increase the use of psychosocial support, and they are not specific to frontline staff. Consequently, future studies and psychosocial support programs in practice should address the identified factors of psychosocial support for hospital staff as a whole.

## Data Availability

The datasets generated and/or analyzed during the current study are not publicly available due to confidentiality of hospitals and participants, but are available from the corresponding author on reasonable request.

## Appendix 1. Demographic and occupational characteristics of the quantitative sample (1514 survey participants)

**Table:**

Category	*n*	%
*Gender*		
Female	1175	77.6
Male	300	19.8
Other	3	0.2
Missing values	36	2.4
*Age*		
< 20 years	7	0.5
20–29 years	221	14.6
30–39 years	275	18.2
40–49 years	310	20.5
50–59 years	474	31.3
> 60 years	198	13.1
Missing values	29	1.9
*Occupational group*		
Management	51	3.4
Medical specialist	87	5.7
Physician resident	43	2.8
Nurse	493	32.5
Support staff	816	53.9
Missing values	24	1.6

## Appendix 2. Codebooks reasons for non-use and important elements

**Table:**

*Reasons for non-use*		
Second order code	First order code	Definition
Unnecessary	Not needed	Revolving around the aspect of deeming psychosocial support not (yet) necessary or not anymore. This includes staff who in hindsight note getting support might have been good
	Other support	Deeming support from others (e.g., family/friends, colleagues, external professionals) or self-support sufficient or better suited
Unsuitable	Not beneficial	Deeming psychosocial support to be not useful or associated with having negative connotations Aspects include stigma (not wanting to appear weak), safety (not feeling safe), confidentiality (lack of confidentiality), negative experience (having had prior negative experiences or mistrusting the hospital)
	Type of support	Revolving around the aspect of wanting a different form of psychosocial support. This includes different channels (face-to-face, chat, telephone), people from different functions (psychologist, social worker, peer, spiritual advisor), different forms (visit departments, in own team, peer support under supervision, one-on-one, consultation hour), different availability (nights, weekends, holidays)
	Lack of time	Revolving around the aspect of not getting psychosocial support due to a lack of time
Unaware	Not sufficiently actively promoted	Revolving around the aspect of support not being sufficiently available or participants being unware of the availability of psychosocial support
	More than COVID-19	Revolving around the aspect of having staff perceiving the support to only be related to COVID topics or focused on departments involved with COVID care
Undeserving	Others need it more	Revolving around the aspect of thinking others need it more, or not wanting to bother the psychosocial support team

**Table:**

Elements that staff deem important regarding the offering of psychosocial support	
Code	Definition
After the pandemic	Includes the aspect of psychosocial support not being offered more structurally after the pandemic
Adjusted to diverse needs	Revolving around the aspect of recognizing that a one size fits all approach doesn’t capture everyone’s needs, and thus offering a more broad spectrum of psychosocial support. Examples include preferences differing regarding who provides the support, how the support is provided, through which channel it’s being offered or when it’s being offered
Accessibility and awareness	Revolving around the aspect of ensuring visibility and an active approach and lowering the threshold for contacting psychosocial support. Examples include good communication and being present at wards
Active supervisors	Suggestions related to the supervisor/team leader. E.g., the supervisor checking up on people, referring them to psychosocial support, identifying staff’s needs
